# Underweight and its associated factors among pediatrics attending HIV Care in South Gondar Zone public health facilities, Northwest Ethiopia, 2021

**DOI:** 10.1186/s12887-022-03630-6

**Published:** 2022-10-05

**Authors:** Chalie Marew Tiruneh, Tigabu Desie Emiru, Nigusie Selomon Tibebu, Moges Wubneh Abate, Adane Birhanu Nigat, Berihun Bantie, Agimasie Tigabu, Amsalu Belete, Belete Gelaw Walle, Getasew Legas

**Affiliations:** 1 Department of Pediatrics and Child Health Nursing, College of Health Sciences, Debre Tabor University, Debre Tabor, Ethiopia; 2 Department of Adult health Nursing, Debre Tabor University, Debre Tabor, Ethiopia; 3 Department of Psychiatry, College of Health Sciences, School of Medicine, Debre Tabor University, Debre Tabor, Ethiopia; 4 Department of Pediatrics and Child Health Nursing, College of Health Science and Medicine, Wolaita Sodo University, Wolaita Sodo, Ethiopia

**Keywords:** Underweight, Pediatrics, HIV/AIDS, South Gondar

## Abstract

**Background:**

Malnutrition associated with HIV infection is a complex condition, with HIV-positive children having a higher mortality rate than HIV-negative children, resulting in significant morbidity and mortality in children. Data from a variety of situations are needed to counter this, but the evidence is limited, especially for the nutritional status of HIV-infected children. Therefore, this study aims to assess the magnitude of underweight and factors associated with it in children receiving antiretroviral therapy.

**Methods:**

An institutional-based cross-sectional study was conducted among HIV-positive children in South Gondar, Northwest Ethiopia. Data were collected using an interviewer-administered questionnaire and anthropometry measurement. Data were coded and entered into Epi-Data Version 3.1 and analyzed using SPSS Version 25. Bivariable and multivariable binary logistic regression models were used to identify factors associated with nutritional status and variables with p-values ˂0.05 in multivariable logistic regression were considered as statistically significant factors.

**Results:**

Of 406 participants, 379 participant were included in the study, which corresponds to a response rate of 93.3%. About one-third (36.4%) of the caregivers were not first relatives and 162 (42.7%) were unable to read and write. Of the study participants, 101 (26.6%) had a CD4 count below the normal threshold. Ninety (23.7%) of those questioned did not follow any nutritional advice from health care workers. In this study, the prevalence of underweight was 106 (28%). In the multivariable analysis being younger age, having low CD4 count, having recurrent diarrhea, and having poor adherence to dietary advice was significantly associated with being underweight.

**Conclusion:**

This study found that the prevalence of underweight among HIV-positive children in south Gondar is significantly high. Therefore, HIV-positive pediatrics who are young, have low CD4 counts, have recurrent diarrhea, and do not adhere to dietary recommendations need to detect and monitor nutritional problems promptly.

## Background

One of the biggest public health issues is the human immunodeficiency virus (HIV) pandemic, which has a number of long- and short-term effects [1]. Around 36.9 million people worldwide were living with HIV as of the end of 2019, with 1.8 million of those being children (age 0–14 years). Ethiopia is one of the nations in sub-Saharan Africa (SSA) that is impacted by the spread of HIV on a worldwide scale. An estimated 56,514 children under the age of 15 had HIV as of the end of 2018[2].

Malnutrition associated with HIV infection is a complex condition, with HIV-positive children having a higher sever acute malnutrition (SAM) related mortality rate than HIV-negative children, resulting in significant morbidity and mortality in children [3]. Compared to their HIV-negative counterparts, HIV-infected children have additional nutritional requirements to ensure normal growth and development and require a high-energy, high-protein, and nutrient-dense diet[4].

It is advised that children with asymptomatic HIV should eat 10% more calories than those without the virus. Children with severe HIV-related problems must increase their daily energy intake by 50–100% until their weight returns. The expense of nutrients to sustain the immune system and avoid muscular wastage is reflected in this higher nutrient requirement. In resource-constrained environments (RLS), where food is scarce, increased nutritional demands are unlikely to be met[4].

For children with HIV, maintaining appropriate nutrition is still a significant difficulty. Antiretroviral therapy (ART) side effects, poor dietary intake, and HIV infection itself are all contributing factors to this issue[5]. Although there have been few studies assessing the nutritional state of children living with HIV in Ethiopia [5–10], the majority of these research have been on the general nutritional status, and some have been on stunting[5–10].

Evidence of the nutritional status of HIV-positive children is essential for policymakers and clinicians to take appropriate action. However, even though the problem is still a major public health problem and a major cause of morbidity and mortality in HIV positive children, studies conducted to assess nutritional status and associated factors in HIV-positive children is still inadequate. Specifically in our study area, no single study is there in this regard. This is the main reason for conducting this study which aims to highlight the extent and associated factors of underweight in HIV-positive children, with implications for improving healthcare worker interventions, ensuring treatment effectiveness, and accelerating the reduction of HIV-related morbidity and mortality in children.

## Methods

### Study area, design, and period

A facility based cross-sectional institutional study was conducted among HIV-positive pediatricians on ART in South Gondar Zone, Ethiopia, from September to November 2021. The capital of the south Gondar zone is Debre Tabor town which is located about 667 km northwest of the capital city of Ethiopia, Addis Ababa, 99 km northeast of Bahirdar city and 50 km east of Lake Tana. It has a latitude and longitude of 11°51′N 38°1′E with an elevation of 2,706 m (8,878 ft) above sea level. The study was conducted in 12 selected healthcare facilities. ART follow-up services are currently being provided for more than 565 children in the study area.

### Study participants, sample size, and sampling technique

All HIV-positive children aged < 15 years taking ART in the South Gondar Zone health facilities were the target group. All HIV-positive pediatrics who had ART follow-ups at the selected hospitals were the study population. Children with incomplete initial medical information, on the other hand, were excluded. In addition, children without caregivers or consenting parents, caregivers with mental health illness, and caregivers with hearing impairment or major medical disorders were all excluded from the study.

The minimum required sample size was determined using a single population proportion formula. To calculate our sample size, the following statistical assumptions were considered: 60.2% proportion (p) of malnutrition from a study conducted in East and West Gojam Zones, Amhara, Northwest, Ethiopia [9]; 5% error rate; 10% non-response rate; and 95% confidence interval (CI).

$$\text{n}=\frac{\left({\text{Z}\text{a}/2}^{2}\text{p}\right(1-\text{p})}{{\left(\text{d}\right)}^{2}} = \frac{\left({1.96)}^{2} 0.6\right(1-0.6)}{{\left(0.05\right)}^{2}} =368.64\tilde369$$

Where, n = the required sample size, Z/2 = standard normal deviation for Type I errors, p = prevalence (0.5) & d = tolerated sampling error margin (0.05).

The calculated sample size was 369. After accounting for a 10% non-response rate, the final sample size of our study was 406. The study was conducted at 12 selected healthcare facilities. From the beginning, a sampling frame was prepared using the patient’s medical registration number from each hospital’s ART registration log. Following that, the total sample sizes were distributed proportionally to each hospital. Finally, using a computer-generated basic random sampling procedure, study participants were chosen from each of the selected healthcare facilities.

### Data collection tool and procedure

The existing ART Clinic Admission and Follow-up Forms of the Ethiopian Federal Ministry of Health served as the basis for the development of the English version Data Extraction Checklist’s. It was translated into Amharic by experts in the English language. By using anthropometry, face to face interviews, and reviews of clinical records, data were gathered by qualified healthcare professionals. For data collectors and supervisors, a one-day training session on study objectives, tool content, and data collection techniques was held. The pre-test was conducted at the Debre Tabor health Center. During data collection, caregivers of underweight children were connected to health care professionals who are in charge of managing the child’s nutritional problem, and all caregivers received nutritional counseling. The assigned supervisors and the principal investigator closely monitored and oversaw the entire data collection process.

### Operational definitions

Under-nutrition: - was defined when the children had either W/H or H/A or W/A z-score <-2 SD of the median value of WHO standard [11, 12].

Stunting: - was defined as children having height-for-age z-score <-2 SD [11, 12].

Wasting: - was defined as children having weight-for-height z-score <-2 SD [11, 12].

Underweight: - was defined as children having weight-for-age z-score <-2 SD [11, 12].

### Data management and statistical analysis

The consistency and completeness of the collected data were checked during data management and analysis. Data were entered into Epi Data version 3.1 and analysis was performed using the Statistics Package for Social Science (SPSS) version 25. The anthropometric measurements were converted to z-scores using the WHO Anthro Plus software version 3.2.2. Frequencies and cross-tabulations are used to check for missing values ​​of variables and to describe the study population in terms of relevant variables. In addition, percentages, proportions, and summary statistics (mean, median) were used to summarize the characteristics of the study population. A binary logistic regression analysis was implemented to assess the association of factors with the outcome variable. Variables with p-values ​​< 0.25 in the bivariate analysis were entered into the final model to control for the effects of confounders and to identify significant factors. The adequacy of the model to fit the outcome variables to the predictors was checked using the Hosmer-Lemeshow goodness-of-fit test. In the multivariate analysis, variables with p-values ​​less than 0.05 at 95% CIs were considered statistically significant factors. Finally, the strength and direction of association were assessed using odds ratios with their respective 95% CIs.

## Results

### Socio-demographic characteristics of study participants

Of 406 participants, 379 participated in the study, which corresponds to a response rate of 93.3%. Almost half of the study participants 190 (50.1%) were male and 131 (34.6%) were from rural areas. One hundred twenty-five (33%) of the study participants were younger than 60 months, while 200 (52.8%) were between 121 and 180 months old. Of the total participants, 138 (36.4%) of the caregivers were not first relatives and 162 (42.7%) of the caregivers were unable to read and write. One hundred and eighty-two children (48%) use an unprotected water source (Table 1).

**Table 1:** Socio demographic characteristics of HIV Positive Pediatrics Attending Care in South Gondar health facilities

Variable	frequency	Percentage		
Ethnicity	Amhara	355	93.7	
	Other	24	6.3	
	Total	379	100.0	
Residency	Urban	248	65.4	
	Rural	131	34.6	
	Total	379	100.0	
Marital status of caretaker	currently not married	289	76.3	
	currently married	90	23.7	
	Total	379	100.0	
Monthly income of caretakers in Birr	< 1500	70	18.5	
	1500 to 3000	120	31.7	
	> 3000	189	49.9	
	Total	379	100.0	
Relationship of caretaker with child	First relative	241	63.6	
	other	138	36.4	
	Total	379	100.0	
Age of child	< 60 month	125	33.0	
	60 to 120 month	54	14.2	
	121 to 180 month	200	52.8	
	Total	379	100.0	
Sex of child	Male	190	50.1	
	Female	189	49.9	
	Total	379	100.0	
Religion of caretaker	Protestant	21	5.5	
	Orthodox	341	90	
	Muslim	17	4.5	
	Total	379	100.0	
Education of caretaker	can read and write	217	57.3	
	not read and write	162	42.7	
	Total	379	100.0	
Occupation of caretakers	Government employed	43	11.3	
	NGO employed	39	10.3	
	Merchant	132	34.8	
	Daily Laborer	144	38.0	
	Other (specify)	21	5.5	
	Total	379	100.0	
Number family	< 4	245	64.6	
	>=4	134	35.4	
	Total	379	100.0	
Birth order	1	91	24.0	
	2	109	28.8	
	3	102	26.9	
	4	77	20.3	
	Total	379	100.0	
Source of water	protected	197	52.0	
	unprotected	182	48.0	
	Total	379	100.0	
Waste disposal system	Closed	217	57.3	
	Open	162	42.7	
	Total	379	100.0	

### Clinically related factors

One hundred seven (28.2%) subjects had WHO clinical stage 4 illness. Of the study participants, 101 (26.6%) had a CD4 count below the normal threshold. Almost two hundred and thirty-eight (62.8%) had another comorbidity. 130 (34.3%) of study participants received antiretroviral treatment less than 60 months duration. One hundred thirty-eight (36.4%) participants had poor medication adherence. 90 (23.7%) of those questioned did not follow any nutritional advice. In this study, the prevalence of underweight was 106 (28%) (Table 2).

**Table 2:** Clinical related factors of HIV Positive Pediatrics Attending Care in South Gondar health facilities

Variable	Category	frequency	Percentage	
WHO stage	Stage 1	141	37.2	
	stage 2	39	10.3	
	stage 3	92	24.3	
	stage 4	107	28.2	
	Total	379	100.0	
CD4 level	Below Normal	101	26.6	
	In Normal range	278	73.4	
	Total	379	100.0	
Have recurrent diarrhea	No	245	64.6	
	Yes	134	35.4	
	Total	379	100.0	
Presence of recurrent oral lesion	No	138	36.4	
	Yes	241	63.6	
	Total	379	100.0	
Any comorbid illness in the last month	No	141	37.2	
	Yes	238	62.8	
	Total	379	100.0	
Month on ART	< 60 month	130	34.3	
	60 to 120 month	71	18.7	
	121 to 180 month	178	47.0	
	Total	379	100.0	
ART drug adherence level	Good	117	30.9	
	Fair	124	32.7	
	Poor	138	36.4	
	Total	379	100.0	
Adherence to nutritional counseling	Yes	289	76.3	
	No	90	23.7	
	Total	279	100	
History of hospital admission	NO	211	55.7	
	Yes	168	44.3	
	Total	379	100.0	
Current weight	Normal weight	273	62.0	
	Under weight	106	28.0	
	Total	379	100.0	

### Factors associated with underweight

In this study, the association between various factors and underweight was assessed. In the bivariate analysis, age, current CD4 count, WHO clinical stage, marital status of caregivers, recurrent diarrhea, source of drinking water, and level of adherence to dietary advice were eligible for an adjustment in the multivariate analysis. In the multivariable analysis, age, current CD4 count, diarrhea, and level of adherence to dietary advice were left to show a statistically significant association with underweight.

In comparison to children older than 120 months, the risks of being underweight were around three times greater (AOR 2.836; 95% CI = 1.668–4.822) in HIV-positive children less than 60 months. HIV-positive children with a CD4 level below the normal threshold had a nearly twofold (AOR = 2.129; 95% CI = 1.233–3.674) increased risk of being underweight (AOR = 2.129; 95% CI = 1.233–3.674) compared to those with a normal CD4 count. Compared to HIV-positive children without diarrhea, children with recurrent diarrhea were twice (AOR = 2.763; 95% CI = 1.581–4.831) as likely to be underweight. Furthermore, HIV-positive pediatrics with poor dietary adherence had a higher likelihood (AOR = 2.290; 95% CI = 1.327–3.954) of being underweight than those with strong adherence to dietary recommendations (Table 3).

**Table 3:** Bivariable and multivariable analysis of HIV Positive Pediatrics Attending Care in South Gondar health facilities

Variable and Category	Underweight		Bi-variable logistic regression analysis		Multi-variable logistic regression analysis	
	Yes N (%)	No N (%)	p-value	COR with 95%CI	AOR with 95% CI	P-Value
Age in month						
< 60	48(45.3%)	77(28.2%)	0.001	2.417(1.470–3.977)	2.836(1.668–4.822)	0.000*
60–120	17(16%)	37 (13.6%)	0.091	1.782(0.913–3.479)		
121–180	41(38.7%)	159(58.2%)		1		
CD4 count						
Below normal	35(33%)	66(24.2%)	0.082	1.546(0.947–2.525)	2.129(1.233–3.674)	0.007*
Normal	71(67%)	207(75.8%)		1		
Marital status						
Not married	33(20.9%)	57(31.1%)	0.036	1.713(1.035–2.836)		
Married	73(79.1%)	216(68.9%)	1			
WHO clinical stage						
Stage 4	38(35.8%)	69 (38.5%)	0.090	1.606(0.929–2.778)		
Stage 3	25(23.6%)	67(11.7%)	0.781	1.088(0.600-1.974)		
Stage 2	7(6.6%)	32(24.5%)	0.328	0.638(0.259–1.571)		
Stage 1	36(36%)	105(25.3%)	1			
Have diarrhea						
Yes	21(19.8%)	113(41.4%)	0.000	2.859(1.674–4.881)	2.763(1.581–4.831)	0.000*
No	85(80.2%)	160(58.6%)	1			
Water source						
Not protected	57(53.8%)	125(45.8%)	0.163	1.377(0.878–2.160)		
Protected	49(46.2%)	148(44.2%)	1			
Have adherence to nutritional counseling						
No	35(33%)	55(20.1%)	0.009	1.954(1.184–3.226)	2.290(1.327–3.954)	0.003*
Yes	71(67%)	218(79.9%)	1			

1- Reference *- significantly associated with the outcome variable

## Discussion

Malnutrition is very common in HIV-infected individuals by affecting food intake, altering digestion and absorption, altering metabolism, and increasing energy needs. At the present study, the prevalence of underweight among children receiving HIV treatment in public health institutions in the south of Gondar zone was 28% ( 95% CI = 23.5–32.5).

The current finding is higher when compared to a study conducted in Tanzania (22.1%)[13]. The discrepancy may result from the difference in sociodemographic factors and study time. However, compared to studies done at Felege Hiwot and Gondar referral hospitals (41.7%)[9], Cameron(37.8%)[14], and India(63%)[15], the prevalence of underweight among pediatrics receiving HIV care in South Gondar Zone public health facilities was lower. The discrepancy in the study period and setting may be the cause of the discrepancy.

In this study, children whose age was less than 60 months were more likely to be underweight. This can be explained by the fact that younger children are unable to communicate their feelings of hunger, the type of feeding they prefer, and the timing of their meals. The prevalence of underweight can therefore be common in this younger age group, especially given the fast disease progression nature in youngsters that leads in greater nutritional demand[16].

In the present study, children who had CD4 levels below the normal threshold were more likely to be underweight. This is consistent with the scientific evidence showing a link between poor dietary status and low CD4 counts [17]. This could be as a result of low CD4 counts in children being exposed to an additional opportunistic illness that causes higher metabolism and decreased appetite[16].

Moreover, HIV-positive pediatrics who had a history of recurrent diarrhea were more likely to be underweight. This is in line with the scientific justification of; recurrent diarrhea decreasing the absorption of nutrients and increasing body starvation, which ends with the decreased weight of the patients[16, 18].

Finding from this study also revealed that the prevalence of underweight was more likely in HIV-positive pediatrics who did not adhere to nutritional counseling. This is also consistent with the scientific evidence that healthy eating can improve overall quality of life, boost immunological function, maximize the effects of antiretroviral therapy, lower the risk of chronic illnesses, and maintain a child’s weight within normal limits. Non-adherence to nutritional counseling may exposes to opportunistic infections (OIs) which further affects their weight [19, 20].

### Strength and limitation of the study

As strength, the study is conducted in a multicenter level and it incorporates health facilities in remote area, at which no study is conducted before this study. In contrast, Despite its remarkable findings, as of any study, the findings of this study should be seen in the light of some limitations that result from the study design, which the study cannot determine temporality of exposure and disease.

## Conclusion

This study found that the prevalence of underweight among HIV-positive children in south Gondar is significantly high. HIV-positive children, who are young, have low CD4 counts, have recurrent diarrhea, and do not adhere to dietary recommendations were significantly associated with underweight. Therefore, concerned organizations should concentrate on the aforementioned variables that are connected with underweight in order to avoid underweight among HIV positive children.

## Data Availability

The data sets used and/or analyzed during the current study are available from the Corresponding author upon reasonable request.

## List of abbreviations

AIDS: Acquired Immune Deficiency Syndrome.
AOR: Adjusted odds ratio.
ART: Antiretroviral Therapy.
CI: Confidence Interval.
COR: Crud odd ratio.
HIV: Human immunodeficiency Virus.
OI: Opportunistic Infection.
SPSS: Statistical Package for Social science.
WHO: World Health Organization.
